# Supplementary material for: A Novel CNN pooling layer for breast cancer segmentation and classification from thermograms
Source: PLoS One. 2022 Oct 21;17(10):e0276523. doi: 10.1371/journal.pone.0276523 (PMC9586394; doi:10.1371/journal.pone.0276523)
Supplement: Attachment — Submitted filename: Comments.docx [file pone.0276523.s002.docx]

**COMMENTS**

The authors propose a strategy for breast cancer diagnosis using thermograms employing pooling layer known as vector pooling block (VPB) which contains two data pathways, focus on extracting features along horizontal and vertical orientations which collect the global and local features. Furthermore, U-Net, AlexNet, ResNet18 and GoogleNet CNN architectures are used for the segmentation and classification. Overall, the work presented in this manuscript is explained well as the authors compare the proposed approach to other existing techniques. Furthermore, the text is clearly written, the methods described clearly, and the results presented in clean figures and easily to understand. Nevertheless, I have some concerns which will improve the quality of the manuscript further. Please see my detailed comments below.

1. The title **“CCN pooling layer”**. What is meant by CCN?
2. What’s the challenge for diagnosing breast cancer at an early stage compared to advanced cancer from the view of image feature and ML algorithms?
3. Explain the role of recent works of U-NET CNN segmentation as well in your work.

- Maqsood, S., Damasevicius, R., & Shah, F. M. (2021, September). An efficient approach for the detection of brain tumor using fuzzy logic and U-NET CNN classification. In International Conference on Computational Science and Its Applications (pp. 105-118). Springer, Cham.
- Du, G., Cao, X., Liang, J., Chen, X., & Zhan, Y. (2020). Medical image segmentation based on u-net: A review. Journal of Imaging Science and Technology, 64, 1-12.
- Maqsood, S., Damaševičius, R., & Maskeliūnas, R. (2022). TTCNN: A Breast Cancer Detection and Classification towards Computer-Aided Diagnosis Using Digital Mammography in Early Stages. Applied Sciences, 12(7), 3273.

4. Explain the proposed method in more detail. The given information of the proposed method is insufficient. Explain the working of Figures 3,4,5.
5. What is the main motivation behind choosing the selected database? If the images are color or grayscale? Please mention.
6. Describe the computer on which the experiments were performed (OS, CPU, RAM, etc.) and programming environment (language) used to implement the method.
7. The proposed method should also be compared with other methods to show the worth, effectiveness and superiority of the work. The work lacks the discussion section.
8. Add the discussion section in your manuscript and explain how and why your results are superior to other. The information provided in the experimental results portion is insufficient.
9. Patient selection criteria should be provided to show what stage of patients the system is effective for, or if it is effective for any stage of patients.
10. Information on the diagnosing doctor should be included to show whether this accuracy can be obtained by any physician.
11. The computation efficiency of the proposed method should be addressed.
12. There is a need for language improvement. I found some grammatical error texts in the manuscript. The language of the paper needs a review.
